# Cardiac MR imaging of cor triatriatum sinister in an elderly man: A rare case report

**DOI:** 10.1016/j.radcr.2024.01.014

**Published:** 2024-01-20

**Authors:** Eppy Buchori, Dian Komala Dewi, Hilman, Pratama Adityabiantoro

**Affiliations:** Radiology Department, Cardiovascular and Interventional Radiology Division, Hasan Sadikin General Hospital, Faculty of Medicine, Padjajaran University, Bandung, Indonesia

**Keywords:** Cor triatriatum, Magnetic resonance imaging, Dyspnea, Elderly

## Abstract

Cor triatriatum sinister an interesting and relatively rare congenital condition in which the left atrium is bisected by a fibromuscular membrane into 2 distinct chambers. Classically, patients are diagnosed at an early age, although in some cases they remain asymptomatic until adulthood and this is mainly due to differences in the degree of obstruction to pulmonary venous return and the presence of associated lesions. However, some patients may remain asymptomatic into adolescence or late adulthood due to incomplete membranes or other favorable circumstances. Although there are only a few cases where cardiac MRI has been used to diagnose cor triatriatum sinister, it is very suitable for making the diagnosis. important to carefully evaluate cases of cor triatriatum sinister, as it resembles the physiology of mitral stenosis. Such cases in elderly individuals accompanied by mitral and tricuspid regurgitation are very rare. We want to present a case of a diagnosed cor triatriatum with mitral and tricuspid regurgitation in a 73-year-old male, who was hospitalized due to signs of heart failure. The diagnosis was made using MRI.

## Introduction

Cor triatriatum sinister is a rare congenital heart defect in which the left atrium is divided into 2 chambers by a fibromuscular membrane. It was first described by Church in 1868 [1]. A posterosuperior chamber receiving the pulmonary veins, and an anteroinferior chamber communicating with the mitral orifice [2]. Many theories for cor triatriatum sinister have been suggested; however, the most widely accepted theory is that it results from a failure of incorporation of the common pulmonary vein into the left atrium during fetal life [1].

Cor triatriatum is typically diagnosed via echocardiography, although other modalities such as CT scan and MRI are also used [3]. MRI is an effective tool for visualizing anatomic abnormalities in individuals with congenital heart disease [4]. We report the case of an elderly man with ischemic dilated cardiomyopathy who presented with new-onset atrial fibrillation with normal ventricular response and acute decompensated heart failure. He underwent an MRI that suggested cor triatriatum sinister.

## Case presentation

A 73-year-old man came to our institution with complaints of dyspnea since about 1 week ago and getting worse since 1 day before entering the hospital. Complaints are felt continuously and do not improve with rest. Complaints improve with sitting position. Complaints accompanied by swelling in both legs and stomach. There is a history of diabetes mellitus, dyslipidemia, and heart disease. He reported taking insulin, furosemide, spironolactone, bisoprolol, and valsartan.

Vital signs were blood pressure of 102/83 mmHg, pulse rate of 78 bpm, irregularly irregular, pulsus deficit (+), heart rate of 92 bpm, respiratory rate of 24 shallow and fast, and oxygen saturation of 99%. On physical exam, he was in no apparent distress but was noted to have a bilateral rhonchi in one-third of the lung fields, no murmurs were detected. There is pitting edema in both lower extremities. His complete blood count, electrolyte levels, and kidney function tests were in normal ranges. An electrocardiogram showed atrial fibrillation (AF) with normoventricular response, RVH, VES Unifocal Frequent. Chest radiograph findings revealed cardiomegaly with signs of pulmonary obstruction, bilateral pleural effusion, and right bronchopneumonia.

Echocardiography demonstrated normal all chamber dimension, concentric LVH, preserved LV systolic function (50%-55% eyeballing) with hypokinetic basal to apical anterolateral, basal to apical anterior, apical anterior normokinetic other segments, LV diastolic dysfunction grade I, normal anatomy and function all valves, low probability of PH, normal RV systolic function, there was no sec and pericardial effusion.

The MRI of the heart was obtained using spin-echo technique. Cardiac MRI showed dilated all chamber with extra atrium at left atrium suggest a cor triatriatum sinistra, there is a 1.10 cm fenestration between left atrium and extra atrium, left ventricle hyperthrophy (eccentric type) with regional wall motion dysfunction of the left ventricle with mid-wall enhancement at basal anteroseptal, inferoseptal, anterior, inferolateral, mid anteroseptal, inferoseptal, and apical septal segments and myocardial edema at mid anteroseptal, inferoseptal, apical septal, and inferior segment of the left ventricle suggest a myocardial infarction at LAD and RCA territories, and also mitral and tricuspid valve regurgitation. The ejection fraction of LV was 31% and RV was 32%.

## Discussion

Cor triatriatum sinistrum is a rare congenital heart anomaly, accounting for only 0.1%-0.4% of cases [5]. Classically, the proximal, or posterior-superior, chamber receives the pulmonary venous drainage, and the distal, or anterior-inferior, chamber contains the mitral valve and the left atrial appendage [6,7]. There is communication between these chambers through one or more openings in the membrane [7].

There are various classification systems for cor triatriatum sinistrum, but Loeffler classified it in 1949 based on the number and size of fenestrations present in the fibromuscular membrane that separates the left atrium. According to this classification, group 1 refers to the absence of any opening, group 2 refers to one or more small openings, and group 3 refers to a single large opening [7]. Patients in group 1 typically display symptoms of left heart obstruction in early infancy, including pulmonary edema. Group 2 and 3 patients are usually asymptomatic, with the condition often detected incidentally in adult populations. In some cases, large fenestrations may become obstructed due to fibrosis and calcification later in life, leading to symptoms such as dyspnea, orthopnea, and hemoptysis [5]. Our patient presented with type 3 morphology, which is typical of nonpediatric cases and less hemodynamically significant.

Cor triatriatum sinistrum is a medical condition where a membrane divides the left atrium of the heart into 2 chambers. The upper chamber, called the upper pulmonary venous chamber, receives the pulmonary venous blood from the pulmonary veins. On the other hand, the lower left atrial chamber is contiguous with the atrioventricular valve and the left atrial appendage arises from it [8]. Due to the obstructive nature of the membrane, there is a pressure gradient created, leading to an increase in pulmonary arterial and venous pressures [3].

Cor triatriatum produces symptoms by causing pulmonary venous obstruction and pressure overloading on the right side of the heart [1]. The presentation of patients can occur in infancy, childhood, or adulthood. This is largely due to variations in the degree of obstruction to pulmonary venous return and the presence of associated lesions [8]. More commonly, however, patients’ symptoms are consistent with left-sided heart failure (ie dyspnea and orthopnea), as would be expected with obstruction at the level of the LA [3].

**Fig. 1:**
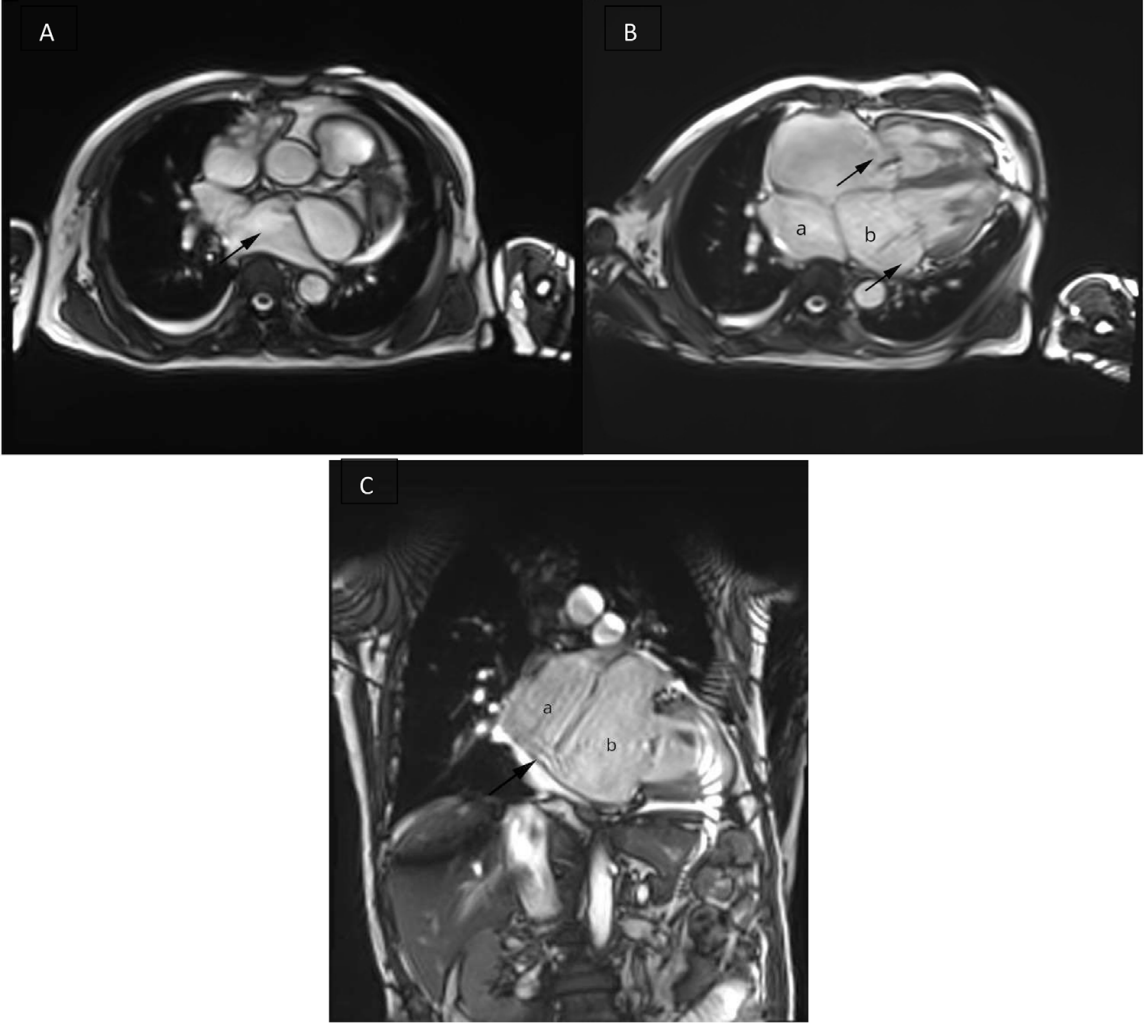
Cardiac MRI. (A) The left atrium branches into the pulmonary artery (arrow). (B) The left atrium has two chambers. Mitral and tricuspid regurgitation was seen (arrow). (C) Slice of 2 chambers. There is a defect between left atrium and extra atrium + 1.10 (arrow).

The most frequent initial symptoms in infants are respiratory distress, cyanosis, recurrent respiratory tract infections, and feeding difficulties, while older patients present with syncope, dyspnea, and hemoptysis [1]. Cor triatriatum is a condition in adults that is often associated with an atrial septal defect and a dilated coronary sinus due to persistent left superior vena cava, as well as a bicuspid aortic valve. The condition may present clinically later on, with the development of atrial fibrillation or mitral regurgitation [2]. Cardioembolic stroke is a regular finding in cor triatriatum sinister and potential mechanisms of thrombus formation include increased prevalence of atrial fibrillation, stagnation of blood flow in the accessory compartment, and paradoxical embolization due to associated ASD [9]. Our patient presents with signs of heart failure that may result from mitral regurgitation, congenital anomaly, or a combination of both. We believe that both causes have led to diffuse hypokinesis and left ventricular dilation Fig. 1.

Echocardiography has been the preferred imaging method for diagnosing cor triatriatum since the 1990s. This technique involves using two-dimensional imaging to visualize the membrane, evaluating turbulent flow with color Doppler, and determining any gradient across the membrane with continuous wave Doppler. In addition, echocardiography can effectively identify any coexisting congenital anomalies [7]. Echocardiography can beautifully depict the interatrial membrane, and the fenestrations within can sometimes be visualized as a jet of increased velocity on color Doppler [5].

Cor triatriatum is typically diagnosed by echocardiography, but other modalities such as computed tomography and MRI are also used. A study compared MRI, echocardiography, and cardiac angiography in evaluating pulmonary venous anomalies, which included cases of cor triatriatum sinister, MRI had a higher detection rate of 95%, compared to 69% for angiography and 38% for echocardiography [3]. Cardiac CT angiography is preferred over cardiac catheterization due to it being noninvasive and providing detailed anatomy [6]. Cardiac CT is a noninvasive method to evaluate coronary artery disease. It is a safer and less expensive alternative to standard coronary angiography. The high spatial and temporal resolutions of cardiac CT enable the acquisition of additional information from single data sets, including cardiac anatomy, wall motion, and myocardial perfusion [7].

Cardiac MRI is used to evaluate hemodynamics, valve disease, and chamber size and function [6]. Cardiac MRI is considered the most reliable method for measuring the size and function of heart chambers. It can be helpful in assessing whether there is an excess load on the right ventricle. Additionally, it can identify the presence of blood clots in the accessory chamber (due to slow blood flow) or rule out the possibility of malignancy [9]. A new pulse sequence called Cine-MRI has been developed to evaluate dynamic cardiac function and hemodynamics in both congenital and acquired heart diseases. The pulse sequence is useful in detecting high velocity flow and turbulent flow, which can be seen as low-signal intensity in contrast to the high-signal intensity that represents normal blood flow [4].

This new technique has some limitations, including limited resolution, partial artifacts, and poor data acquisition due to heart rhythm problems. Furthermore, the images cannot be obtained in real-time. Nevertheless, cine-MRI, like standard spin-echo MRI, can scan the entire left atrium without any hindrances from anatomic obstacles. It is also suitable for evaluating the hemodynamics of this disease [4].

Treatment in CTS depends on the severity of the patient's symptoms. Incidentally detected lesions in asymptomatic patients do not warrant any active management. Significant dyspnea and pulmonary congestion are treated by diuretics, digoxin, and preload reduction, while surgical intervention is reserved for highly symptomatic patients with significant obstruction [5].

## Conclusion

Cor triatriatum sinister is a congenital heart disease that is rare but important. In adults, it is a rare condition that can cause a wide range of clinical issues, including mitral regurgitation and atrial fibrillation. Detecting and distinguishing it from other congenital abnormalities requires careful examination. MRI is an effective way to image congenital heart disease. In this case, MRI provided excellent preoperative imaging of the intra-atrial membrane and obstructed flow in the accessory chamber of cor triatriatum. Our case is an example of a rare congenital anomaly that was diagnosed using noninvasive cardiac MR imaging.

## Patient consent

Written informed consent has been received from the patient.
